# 开放性实验:QuEChERS联合荧光衍生法检测莠去津及其对酶活性的影响

**DOI:** 10.3724/SP.J.1123.2024.06010

**Published:** 2025-04-08

**Authors:** Xinghua HUANG, Yiyao HUANG, Wu GAO, Yida ZHANG, Xiaoyan LIU, Haixia ZHANG

**Affiliations:** 兰州大学化学化工学院,甘肃 兰州 730000; College of Chemistry and Chemical Engineering, Lanzhou University, Lanzhou 730000, China

**Keywords:** 开放性实验, QuEChERS, 荧光检测, 莠去津, 过氧化氢酶, 分子对接, open experiment, QuEChERS, fluorescence detection, atrazine, catalase, molecular docking

## Abstract

莠去津是三嗪类农药的代表,其不仅能干扰生物正常生理活动,还可通过诱发氧化应激对植物体造成明显的毒性作用。因此,莠去津的定量检测和毒性机理研究对保证食品安全和维持植物正常生长十分重要。本实验建立了一种QuEChERS和荧光衍生结合的策略,实现了实际样品中莠去津的快速灵敏定量检测。该策略具有选择性好、灵敏度高和操作简单的优点。此外,通过酶活性考察与分子对接探究了莠去津对过氧化氢酶活性的影响,实现了莠去津的毒性机理探究和分子层面验证。通过两部分工作的有机结合,回答了“为什么测”“怎么测”“原理是什么”的问题。本实验涉及样品前处理、光谱和分子对接的相关知识,使用的仪器在高校中普及程度较高,十分适合作为开放性实验进行实验教学。同时,本实验力图通过全方位实验操作、理论结合和应用研究,使学生充分了解分离材料、荧光衍生试剂的选择与应用、分析仪器的检测原理、酶的使用和分子对接相关原理及应用范围。该实验不仅有助于增强学生关注民生、注重分析化学理念和操作规范意识,还能指导学生初步掌握科学研究的思维方法并提升解决实际问题的综合能力。

## Abstract (en)

Atrazine is a triazine pesticide that interferes with normal physiological activities, induces oxidative stress, and is significantly toxic to plants. Therefore, the ability to quantitatively detect atrazine and study the mechanisms responsible for its toxicity are crucial for ensuring food safety and maintaining plant growth. Herein, a method was developed by QuEChERS combined with fluorescence derivatization to rapidly, sensitively, and quantitatively detect atrazine in real samples. This approach offers excellent selectivity and high sensitivity, and is simple to operate. In addition, we also investigated the effect of atrazine on catalase activity through enzyme-activity assays and molecular-docking studies to explore the mechanism responsible for the toxicity of atrazine on the molecular level. This experiment involves knowledge related to sample pretreatment, spectroscopy, and molecular docking, while the instruments used are widely available in colleges and universities and are very suitable for teaching open experiments. This experiment aims to provide students with a comprehensive understanding of experimental operations integrated with theory, as well as applied research aimed at helping students understand the use of separation materials, the selection and application of fluorescent derivatization reagents, the principles of analytical instruments, the use of enzymes, and the principles of molecular docking. This experiment is expected to enhance students’ awareness of people’s livelihoods, analytical chemistry concepts, and operational standards, which will guide the development of their scientific research thinking and improve their abilities to solve real-world problems.

莠去津(atrazine)又名阿特拉津,是一种三嗪类除草剂,能通过竞争性占据质体醌在D1蛋白上的结合位点,阻碍光系统的电子有效传递,进而使植物光合作用无法正常进行。同时,这些过剩的电子又会导致三线态叶绿素和单线态氧的形成,损伤细胞内的生物活性分子,进而杀死细胞,实现除草的功能^[1⇓-3]^。由于成本较低、除草效果优良,莠去津已被广泛用于各类粮食作物(例如玉米、高粱、甘蔗等)的田、牧场及果园出苗前和出苗后阔叶杂草、禾本科杂草和藻类的去除^[4]^,一举成为全球第二常用的除草剂^[5]^。然而,多个研究表明,作为一种环境内分泌干扰物(environmental endocrine disruptor),莠去津不仅会影响肠道微生物^[6,7]^、生殖功能^[8⇓⇓-11]^、器官发育^[12]^、免疫系统^[13]^和神经系统^[14]^等,其还可通过生物累积造成内分泌系统毒性,甚至引发癌症^[15⇓-17]^。因此,莠去津的检测对于维护国民生命健康和食品安全十分重要。

在现有检测技术中,色谱-质谱联用技术因定量准确、灵敏度高且能同时检测多个组分,成为莠去津等物质定量检测常用的方法之一^[18,19]^。然而,该方法通常具有时间成本高、前处理复杂和易受样品基质影响等不足。因此,为了避免上述所提到的问题,对实际样品进行一定的前处理,以减少基质效应的影响和缩短检测时间是十分必要的。根据2020版《中华人民共和国药典》,在农药残留分析中,主要有三类样品前处理方法,即直接提取法、QuEChERS法和固相萃取法。其中,QuEChERS法因具有快速、简便、经济、高效、耐用、安全的特点,从2003年起,就广泛应用于各种物质的前处理^[20⇓-22]^,并已成为分析化学家协会(AOAC 2007.01)和欧洲标准化委员会(EN 15662-2008)的官方指定标准。同时,在国家标准中,QuEChERS法也用于莠去津的定量测定(GB 23200.113-2018)。此外,该方法还可以灵活地与其他分析检测方法联用,实现复杂体系中目标物的快速定量检测。因此,结合QuEChERS法发展一种选择性好、成本低、灵敏度高且操作简便的莠去津定量检测方法非常重要。

莠去津还会对人体和动物造成不同程度的伤害。莠去津可通过诱发氧化应激对植物造成明显的毒性作用,并使其出现不同程度的抗毒性反应。因此,莠去津的毒性研究和机理考察对于深入分析其生理功能和保证动植物正常生理活动十分重要。研究表明,植物会对莠去津产生的毒性做出抗毒性调节,而其抗性很大程度上来自酶类抗氧化防御系统的作用^[23⇓-25]^,该系统常见的参与者包括过氧化氢酶(catalase, CAT)等。例如,随着莠去津浓度的增加,玉米机体中的过氧化氢含量增加,为了应对氧化应激,植物体中过氧化氢酶等的含量和活性显著增加以清除机体中的活性氧^[26,27]^。此外,美洲狼尾草长期(>28天)暴露于一定浓度的莠去津时,植物体内过氧化氢酶等的活性会发生变化^[23]^。所以,研究莠去津引起的抗氧化酶活性增强机制,可以为检测莠去津与其生理功能研究提供理论依据。

本实验建立了一种QuEChERS和荧光衍生结合的策略,实现了实际样品中莠去津的快速灵敏定量检测。通过酶活性考察与分子对接探究了莠去津对过氧化氢酶活性的影响。在该开放性实验的过程中,学生通过查阅文献,首先可以了解莠去津的样品前处理方法、荧光检测法和酶活性检测法的优缺点和相关理论知识,并设计相关实验和操作方法,实现莠去津的快速分离、定量检测和酶活性研究。其次,通过分子对接从理论计算层面验证了实验的相关结果和物质之间的相互作用。在实验过程中,学生不仅能将溶液配制、前处理、化学衍生方法和常用分析仪器的基本操作有机结合起来,还能亲身操作相关分析仪器、计算化学软件和生物化学的相关试剂设备,并进行实验数据的处理和分析。这种方式将实验技能训练与科学研究方法结合起来,有助于培养学生的综合实验能力和创新思维。

## 1 实验部分

### 1.1 莠去津定量方法及原理

莠去津自身没有荧光,使用2-氰乙酰胺作为荧光报告基团在碱性介质和高温条件下进行莠去津的衍生化,得到了具有荧光信号的荧光产物。

该衍生化反应的机理(图1)如下:氨从氰基乙酰胺亚甲基上夺取一个质子形成碳负离子中间体,中间体进攻莠去津中三嗪环上与氯原子相连的碳,生成的中间体经过重排得到具有荧光的化合物^[28]^。

**图1:**
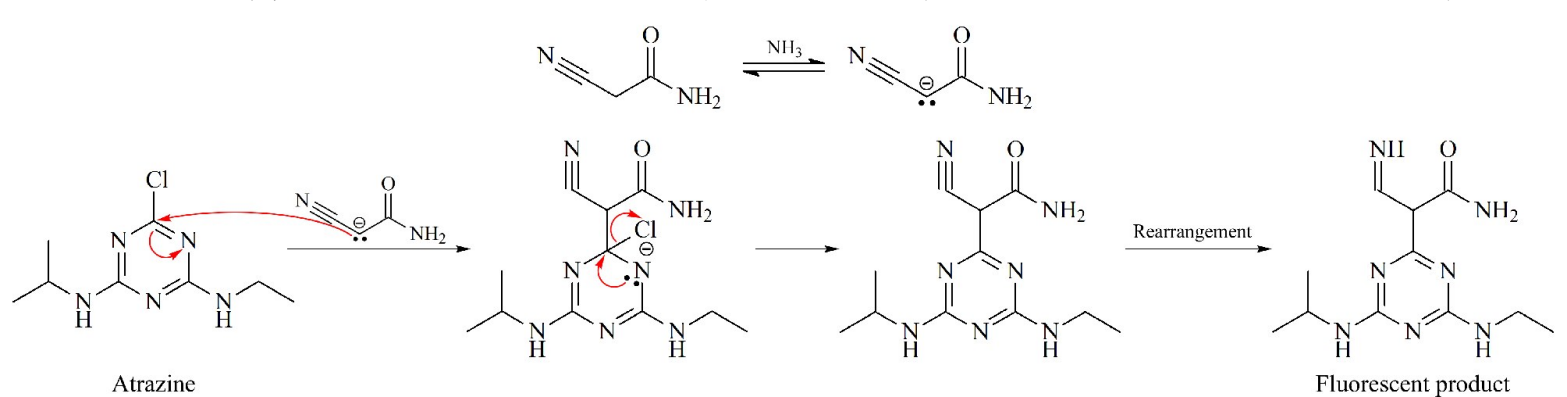
莠去津荧光衍生反应示意图

### 1.2 主要仪器与试剂

紫外光谱仪(T700,北京普析通用仪器有限责任公司);荧光光谱仪(RF-5301 PC,岛津企业管理(中国)有限公司);离心机(H1850,湖南湘仪实验室仪器开发有限公司)。

氢氧化钠和氯化钠(天津市大茂化学试剂厂);柠檬酸二钠(倍半水合物)(>98%,梯希爱(上海)化成工业发展有限公司); PSA和GCB(40~60 μm,天津市津杨化工厂);莠去津(97%,天津市希恩斯生化科技有限公司); 2-氰乙酰胺和过氧化氢酶(≥200000 U/g,上海阿拉丁生化科技股份有限公司);磷酸二氢钾、硫酸镁(≥98.0%,国药集团化学试剂有限公司);无水甲醇、乙腈和氨水(成都市科隆化学品有限公司)。

将0.05 g莠去津标准品溶解在适量无水甲醇中,用无水甲醇定容至100 mL,得到500 μg/mL莠去津标准储备液;用甲醇稀释莠去津标准储备液,配制不同质量浓度的莠去津标准溶液。

取100 μL 200000 U/g的过氧化氢酶原液,用超纯水定容至10 mL容量瓶中,配制得到酶储备液;取500 μL酶储备液,用超纯水定容至10 mL容量瓶中,配制得到酶反应液。

### 1.3 实验步骤

#### 1.3.1 样品前处理

称取4 g硫酸镁、1 g氯化钠、1 g柠檬酸钠、0.5 g柠檬酸二钠(倍半水合物)分别置于10 mL离心管中,得QuEChERS盐包;称取1200 mg硫酸镁、200 mg PSA、20 mg GCB分别置于50 mL离心管中,得QuEChERS净化管。称取50 g购于本地超市的葡萄样品并充分研磨后,将10 g葡萄浆液置于50 mL离心管中。分别在不同离心管中加入不同体积(0、50、125、250 μL)的20 μg/mL莠去津标准溶液,标注相应编号。再用移液枪向各离心管中加入10 mL乙腈,置于涡旋振荡仪上,振荡30 s,静置10 min后得到萃取液。

向盛有葡萄浆液的50 mL离心管中加入上述QuEChERS盐包和1颗陶瓷均质子,盖上离心管盖,置于涡旋振荡仪上,振荡1 min,置于离心机中,于4200 r/min下离心5 min。然后用移液枪吸取8 mL上清液并置于净化管中,于涡旋振荡仪上振荡1 min,并再次将其置于离心机中,于4200 r/min下离心5 min。离心结束后,用5 mL注射器吸取5 mL上清液,用过滤头过滤至10 mL离心管中,置于氮吹仪上,于50 ℃水浴氮吹至干燥。

在进行实际样品加标回收率检测时,在氮吹后的实际样品提取物中加入3 mL 1.0% 2-氰乙酰胺溶液和1 mL 12.0 mol/L氨水,并根据定量后的结果计算加标回收率。

#### 1.3.2 莠去津的定量检测

向50 mL锥形瓶中加入3 mL 1.0% 2-氰乙酰胺溶液、1 mL 12.0 mol/L氨水和不同体积(0、12.5、25、50、250、625 μL)的20 μg/mL莠去津溶液。随后将锥形瓶置于沸水浴中(通风橱内)敞口加热15 min。加热结束后,取出锥形瓶,冷却至室温。将冷却好的溶液转移至10 mL比色管中,用蒸馏水润洗锥形瓶2~3次,将润洗液合并至比色管中,用蒸馏水定容至5 mL。设置激发波长*λ*_ex_为330 nm,发射波长*λ*_em_为376 nm,狭缝宽度分别为5 nm(激发)和10 nm(发射),分别测量各溶液的荧光强度(FI)。以各溶液中莠去津含量为横坐标,荧光强度为纵坐标绘制工作曲线。

#### 1.3.3 过氧化氢酶活性研究

进行过氧化氢酶研究时,向5 mL离心管中依次加入相应体积的5 μg/mL莠去津溶液(0、0、50、100、200、500、1000 μL)、50 mmol/L磷酸缓冲液(2800、2700、2650、2600、2500、2200、1700 μL)及100 μL酶反应液(第一组除外),并将离心管置于25 ℃恒温水浴中,恒温孵育40 min。孵育结束后,在各离心管中加入200 μL H_2_O_2_溶液,反应5 min后,在紫外分光光度计上测定240 nm处的吸光度,绘制过氧化氢酶酶活性(以Δ*A_n_=A*_1_*-A_n_*来表示)随莠去津浓度变化的曲线。其中*A*_1_为体系的初始紫外吸收强度,*A_n_*为酶孵育后体系的紫外吸收强度,Δ*A_n_*为体系的初始紫外吸收强度与酶孵育后体系的紫外吸收强度的差值。

#### 1.3.4 分子对接实验

使用AutoDockTools软件指定莠去津的扭转和可旋转键。后从蛋白质数据库中(https://www.rcsb)下载过氧化氢酶的晶体结构,并用PyMOL软件除去其中的原始配体、水和其他原子,再用AutoDockTools软件给过氧化氢酶添加氢原子,计算总电荷。使用AutoDock Vina进行莠去津和酶的半柔性对接。选择莠去津的最佳构象与酶进行对接结果分析,用Discovery Studio 4.5和PyMOL对对接结果进行可视化分析。

## 2 结果与讨论

### 2.1 莠去津含量的荧光线性相关性

荧光强度检测结果可以看出,衍生后的莠去津具有非常好的荧光线性相关性,其在376 nm处的荧光强度随莠去津浓度的增加而增强,在0.05~2.5 μg/mL范围内,所得线性方程为*y*=65.797*x*+25.493, *R*^2^=0.9999(其中*x*是莠去津的含量,单位为μg, *y*为荧光强度的信号值,*R*^2^为线性相关系数)。此外,本方法检出限(LOD, *S/N*>3)为0.045 ng/mL,定量限(LOQ, *S/N*>10)为0.151 ng/mL,定量限满足国标中规定的莠去津最低残留限量检测需求,因此可用于蔬果、谷物等食品中莠去津的定量检测。

### 2.2 实际样品检测

用所建立的方法进行一批次葡萄样品中莠去津的定量检测,并进行了加标回收试验(表1)。根据所建立的方法,在葡萄样品中未检出莠去津,得到加标回收率为101.1%~111.7%,说明所建立的方法具有一定的可靠性。

**表1:** 实际样品测定及加标回收结果(*n*=3)

Sample	Spiked content/ (mg/kg)	Atrazine content/ (mg/kg)	Recovery/ %
1	0	-	-
2	0.02	0.025	111.7
3	0.05	0.056	106.7
4	0.10	0.104	101.1

### 2.3 过氧化氢酶活性研究

为了研究莠去津诱发氧化应激对植物造成的明显毒性作用,我们使用过氧化氢酶来研究其与酶活性的关系。结果如图2所示,过氧化氢酶的活性随莠去津含量的升高而逐步降低。出现这种变化的原因是由于过氧化氢在240 nm的波长下有强烈的吸收,而过氧化氢酶能使过氧化氢分解使其浓度降低,吸光度下降。因此,通过测量一定时间内过氧化氢酶在240 nm处的吸光度变化来检测其活性。

**图2:**
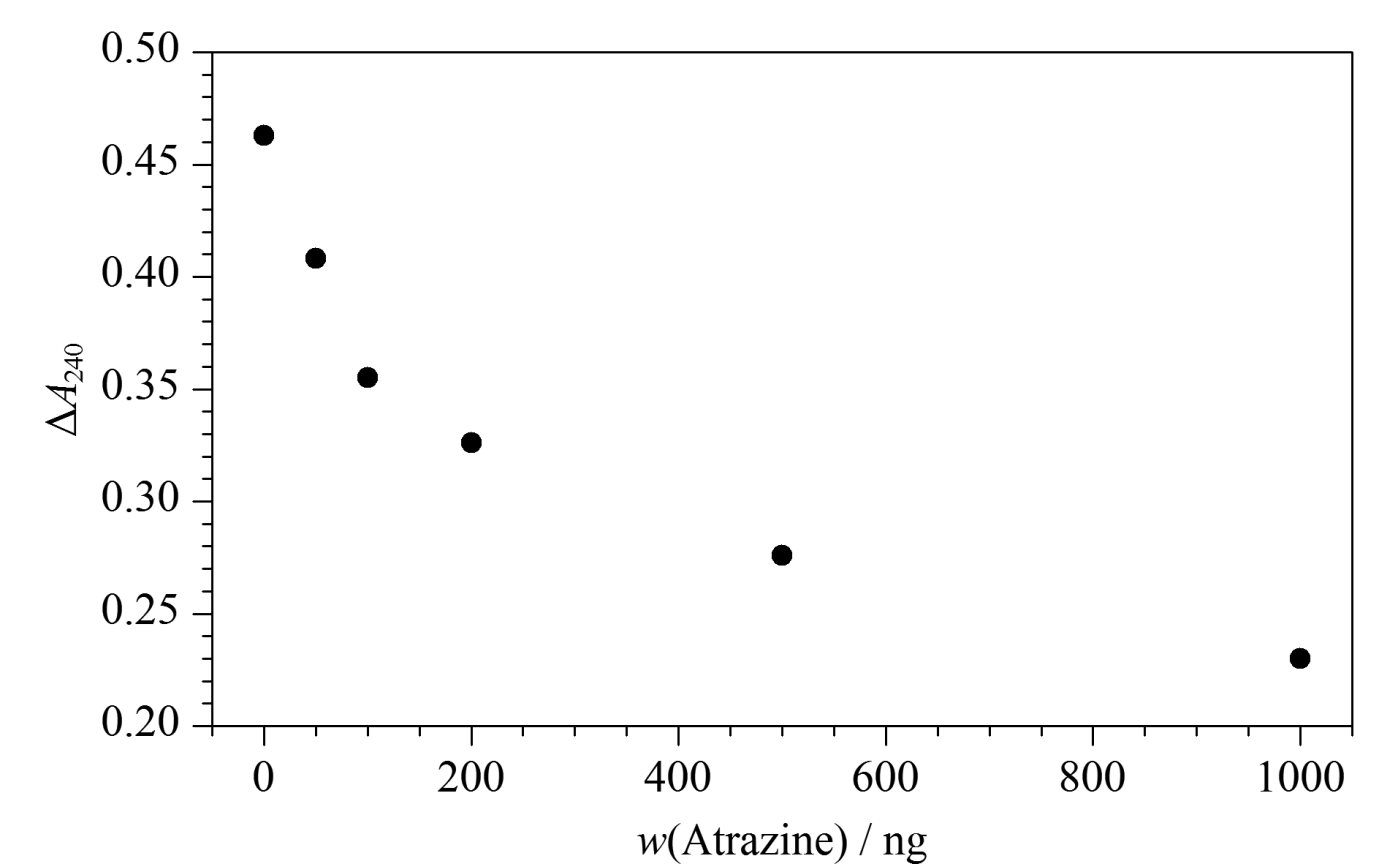
莠去津总量与过氧化氢酶活性的关系

为了进一步验证该结果,我们通过分子对接模拟了过氧化氢酶与莠去津的相互作用。根据分子对接结果,HEM(过氧化氢酶的天然配体)的对接评分为-14.779,而莠去津的对接评分为-8.074。与过氧化氢酶的天然配体HEM相比,过氧化氢酶与莠去津有更强的相互作用。因此,过氧化氢酶活性的降低是由于结合位点被莠去津占据造成的。此外,我们选择了莠去津的最佳构象与酶进行了可视化分析,结果如图3所示,二者的结合位点在天冬酰胺残基处与莠去津具有强的氢键相互作用。该结果可从理论计算层面验证莠去津与过氧化氢酶的相互作用。

**图3:**
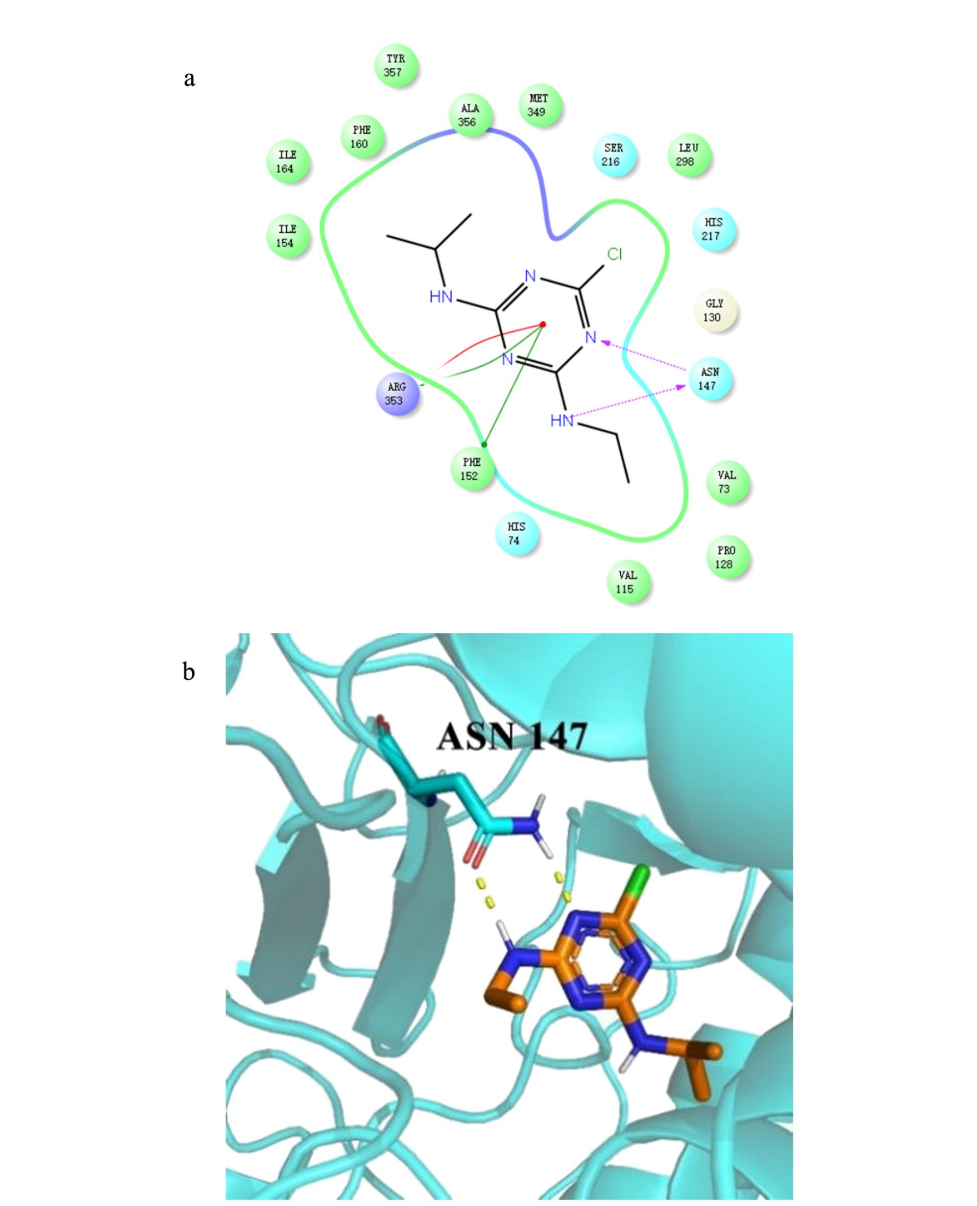
莠去津与过氧化氢酶对接的(a)二维和(b)三维区域图

## 3 实验开展

### 3.1 实验时间安排与反馈

该实验的开展,可分为以下几个部分:试剂的配制(0.5学时)、定量方法的建立(1学时)、样品前处理(1.5学时)、实际样品检测(1学时)、过氧化氢酶活性研究(1.5学时)和实验结果讨论与总结(1.5学时)。如需进一步拓展实验内容,可结合分子对接实验与结果分析(1学时)。实验使用的仪器在高校中普及程度高,实验成本低(每人约9元),符合本科生教学的要求。

在实验结束后,调研结果显示学生们对该创新实验充满兴趣。学生们表示,QuEChERS和荧光衍生结合的策略具有强的创新性,可为其他目标物质检测提供新的思路。使用分子对接从理论角度进行机理分析也具有新颖性,是学科发展和研究的前沿内容。此外,通过调研前沿文献,学生们开阔了视野,并且数据分析能力和仪器操作技能等都得到了提高。他们的实验设计能力和创新思维也得到了锻炼。这些反馈表明,将学科前沿知识引入实验教学,对提升学生的综合能力非常有益。

### 3.2 实验注意事项

要在规定学时内完成本实验,需要注意以下事项:(1)在进行QuEChERS处理时需要注意氮吹仪出气量的大小;(2)进行莠去津的衍生时需要注意混合物的混匀情况;(3)进行紫外吸光度检测时需要将比色皿清洗干净;(4)实验涉及有机反应,需要进行合理的实验防护;(5)在使用大型仪器时应注意操作规范。

## 4 结论

本创新实验以农残为主题,建立了一种QuEChERS和荧光衍生结合的策略,实现了实际样品中莠去津的快速灵敏定量检测,并通过酶活性考察与分子对接探究了莠去津对过氧化氢酶活性的影响,研究了农残与植物毒性的内在联系。通过实验,学生能够了解紫外和荧光光谱基本知识、衍生和分离富集等样品前处理操作与原理、酶活测定和酶与物质的相互作用基本研究方法(实验探索结合理论计算)等;了解莠去津除草的生物学原理及植物应激产生抗性的生物学机制等跨学科信息;认识并加深“严把农残关”、实验相关操作、理论与实际相结合等理念。通过本实验,同学们完成并了解了实际样品中农残测定的全过程,增强了团队协作能力;不仅深刻意识到农残检测的重要性,促进了理论知识与分析测试实践之间的有效互动,还提高了解决实际问题的能力及实践创新能力。最终达到激发学生对科学研究的兴趣、提高学生的动手操作能力、培养学生勤于思考的科学精神和全面提升创新能力和综合素质的目的,更有助于学生全面发展,为将来的科研工作打下坚实基础。
